# Disease severity drives risk of venous thrombotic events in women with sickle cell disease in a single-center retrospective study

**DOI:** 10.1016/j.rpth.2024.102471

**Published:** 2024-06-10

**Authors:** Jennifer Light, Christina M. Abrams, Anton Ilich, Shuai Huang, Hongtu Zhu, Jacquelyn Baskin-Miller, Erica M. Sparkenbaugh

**Affiliations:** 1 Division of Hematology, Department of Medicine, Blood Research Center, University of North Carolina at Chapel Hill, Chapel Hill, North Carolina, USA; 2 Division of Hematology/Oncology, Department of Pediatrics, University of North Carolina at Chapel Hill, Chapel Hill, North Carolina, USA; 3 Division of Pediatric Hematology and Oncology, University of Illinois Chicago at Peoria, Peoria, Illinois, USA; 4 Division of Hematology/Oncology, Department of Pediatrics, Medical University of South Carolina, Charleston, South Carolina, USA; 5 Department of Biostatistics, University of North Carolina at Chapel Hill, Chapel Hill, North Carolina, USA

**Keywords:** contraception, estrogen-containing contraception, progesterone-only contraception, pulmonary embolism, sickle cell disease, venous thrombosis, women’s health

## Abstract

**Background:**

Estrogen-containing hormonal contraception (HC) is a well-established risk factor for venous thromboembolism (VTE). Women with sickle cell disease (SCD) also have an increased risk of VTE. However, it is unknown if exposure to HC exacerbates the risk of VTE in women with SCD.

**Objectives:**

Assess the impact of HC on VTE risk in women with SCD and explore additional risk factors contributing to VTE development.

**Methods:**

We analyzed a retrospective cohort of women of reproductive age (15-49 years) with SCD at the University of North Carolina from 2010 to 2022.

**Results:**

We identified 370 women with SCD, and 93 (25.1%) had a history of VTE. Among 219 women exposed to HC, 38 of 184 (20.6%) had a VTE while actively using HC, whereas 20 of 151 (13.2%) women never exposed to HC had a VTE. Of the patients exposed to HC, 64 of 184 (34.7%) were on estrogen-containing HC, with 120 of 184 (65.3%) using progestin-only formulations. Cox regression analysis found that progestin-only formulations increased VTE risk (hazard ratio: 2.03; 95% CI: 1.107-3.726, *P* < .05). However, when accounting for disease severity, the association between progestin-only treatment and VTE risk was not significant. Indeed, a nuanced analysis revealed that both severe (odds ratio: 11.79; 95% CI: 5.14-27.06; *P* < .001) and moderate (odds ratio: 4.37; 95% CI: 1.77-10.76; *P* = .001) disease increased risk compared with mild disease. Neither genotype nor hydroxyurea use influenced VTE risk.

**Conclusion:**

Overall, we found that increased thrombotic risk is more likely influenced by disease status than HC exposure and should play a role in shared decision-making with patients.

## Essentials

• Women with sickle cell disease are at increased risk of thrombosis.
• Venous thrombosis and contraception use were analyzed in 370 women with sickle cell disease from one institution.
• Contraception status was not associated with thrombosis.
• Severe disease was associated with increased thrombosis risk.

## 1 Introduction

In sickle cell disease (SCD), a genetic point mutation changes a glutamine to valine in the β-globin chain, generating sickle hemoglobin (HbS). Upon deoxygenation, HbS polymerizes within erythrocytes and causes cells to become rigid and sickle-shaped, leading to hemolytic anemia and blockage of the microvasculature called vaso-occlusive events [1,2]. These primary pathologies mediate endothelial dysfunction, painful episodes, and end-organ damage, all contributing to the disease phenotype seen in individuals with SCD [1]. A broadened understanding of the disease now implicates the coagulation cascade in its pathophysiology [[3], [4], [5], [6]], and individuals with SCD are at increased risk of venous thromboembolism (VTE) [[7], [8], [9]]. The incidence of VTE in individuals with SCD is 14% to 25%, with a median age at first event between 25 and 30 years [[9], [10], [11], [12], [13]]. This translates to roughly 4 times the risk of VTE in the general population and is similar to other thrombophilias such as antithrombin deficiency (5-10 times risk) and factor (F)V Leiden (3-5 times risk) [[14], [15], [16], [17]]. Notably, women with SCD exhibit an increased risk of VTE compared with men, with a hazard ratio of 1.18 to 1.22 and a reported odds ratio (OR) of 1.9 [8,18,19]. Known risk factors for VTE in women with SCD include disease severity (assessed by frequency of hospitalization), pulmonary hypertension, avascular necrosis, acute chest syndrome, and vaso-occlusive crisis [8,18,19]. Pregnancy is a recognized risk factor for VTE in general, and this risk is substantially amplified in women with SCD [20], but the increased risk of VTE has been found to be independent of pregnancy [21,22]. This underscores the need to explore additional factors contributing to VTE in this specific population.

Hormonal contraception (HC), particularly estrogen-containing HC, increases the risk of VTE by about 2.5-fold in the general population [[23], [24], [25]]. Combined oral contraception synergistically increases the risk of VTE in women with antithrombin deficiency and FV Leiden by approximately 20 to 30 fold [17]. Considering this and the higher vulnerability of women with SCD [8,18,19], many factors must be discussed with women when initiating a contraceptive method, including the risk of VTE [26]. According to the 2016 Medical Eligibility Criteria for Contraceptive Use Guidelines, there are currently no restrictions on progesterone-only contraception (POC) use in SCD patients. These guidelines consider estrogen-containing HC safe in this population because the advantages are thought to outweigh the risks [27]. Sickle cell providers have valid concerns that estrogen-containing HC might compound the risk of VTE in SCD; however, many clinicians prescribe POC to their patients instead of estrogen-containing HC [26,28]. Recent meta-analyses have identified that some POCs, including depot medroxyprogesterone acetate (DMPA), have an elevated risk of VTE in the general population [29] and women with inherited thrombophilias [15]. Whether there is an additive or synergistic risk of VTE in women with SCD on combined oral contraception or POC has been minimally studied [19,26,30,31].

The primary objective of this study is to assess the impact of HC on VTE risk in women with SCD. We also explore additional risk factors contributing to VTE development. This research aims to discern the VTE risks associated with different HC methods, facilitating informed decision-making for patients based on their age, lifestyle, and reproductive goals [32,33].

## 2 Methods

**Figure 1:**
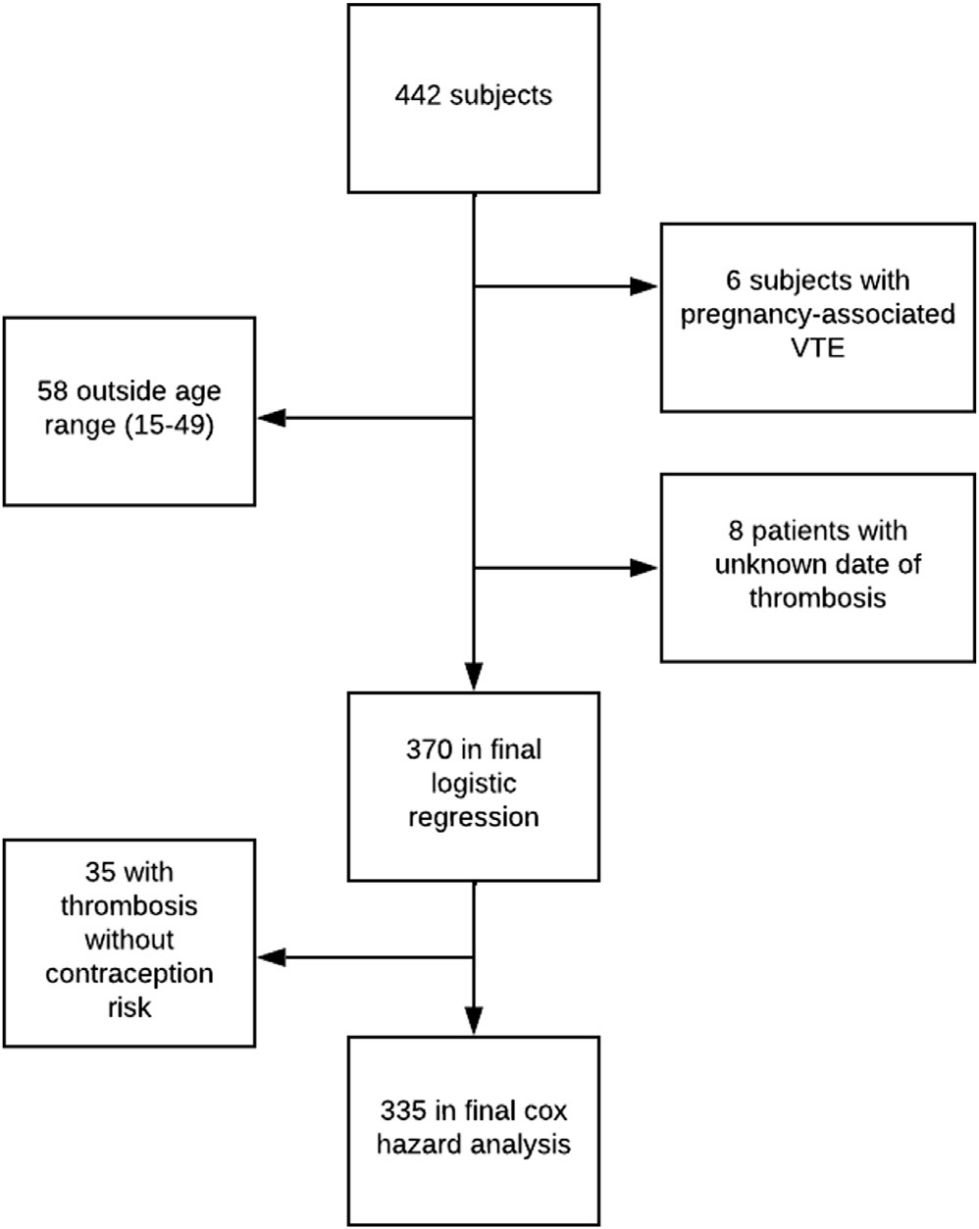
CONSORT diagram depicting the process of exclusions. The initial subjects were obtained through data mining for inclusion criteria.

A retrospective cross-sectional study was performed, using data collected and reviewed over a 6-month time span from records for all female patients with a diagnosis of SCD seen at the University of North Carolina (UNC) between 2010 and 2022. This study was approved by the Institutional Review Board of UNC, and consent was not required for anonymized chart review. Exclusion criteria were sickle cell trait or hemoglobin AA status. Data were initially extracted by looking for inclusion criteria as follows: females between the ages of 15 and 49 years [34] with a confirmed diagnosis of SCD. SCD and VTE, including deep vein thrombosis (DVT) and pulmonary embolism, were defined with ICD9 or ICD10 codes (Supplementary Methods). VTE was identified during clinical care. The codes were confirmed by locating the event date in the chart and determining that there were correlating imaging findings or 2 discrete notations of the diagnosis in the chart to limit the possibility of errors being copied forward [35]. Data regarding the development of thrombosis at any time in the record, presence, and type of contraception, presence and type of VTE (DVT or PE), and confounders such as age, SCD genotype, severity of SCD and associated complications, and other major VTE risk factors were also recorded. Contraception was recorded both if the subject was ever on contraception and if they were on contraception at the time of VTE. If multiple VTEs were noted in the record, the date of the first thrombosis was recorded as the event. HC was defined as the following categories: combined oral contraceptives, estrogen-containing patches or rings, depot progesterone, implanted progesterone, progesterone-only pills, and progesterone intrauterine device (IUD). As there were very few in several of the categories, combined oral contraceptives and patch/ring were pooled as there were only 3 subjects in the patch/ring group and all were estrogen-containing HC. Pregnancy was recorded in 2 ways: if the subject had ever been pregnant and if they were pregnant at the time of thrombosis. The subjects who had missing data for the main outcome of thrombosis and exposure to contraception were imputed to not have thrombosis or contraception. Additionally, 6 women were removed due to pregnancy-associated thrombosis, and 8 were removed because we could not determine the date of VTE, resulting in a final sample size of 370 for initial analysis. For Cox regression analysis, the 35 individuals with VTE who were not on HC at the time of event were removed to allow for analysis without those confounders (Figure 1).

Of the 370 women, subjects were defined as being on HC at the time of VTE based on the type of contraception. For oral contraception (including progesterone-only or combined), a subject was defined as being on HC if prescribed prior to VTE, and there was no indication that they had stopped in the last 30 days prior to VTE, defined as a prescription that was filled. For implantable devices (such as IUD or contraceptive implant), the individual was considered to have contraception in place if it was implanted prior to VTE and there was no indication of removal. Lastly, for those on DMPA, those who started DMPA before VTE and, according to records, were still receiving injections were considered exposed. Central line status was defined as ever having a central line for more than 24 hours to rule out individuals that had a central line for only an exchange transfusion. Central lines included portacath, tunneled catheters, or untunneled apheresis catheters.

The severity of SCD was defined as mild, moderate, or severe using a scoring system reported previously [36], omitting VTE since it was the focus of this study. In addition, we used a simplified severity classification determined by the average number of unplanned VOC-related emergency department visits per year (mild: 0-1; moderate: 2-5; severe: >5) [37].

### 2.1 Statistical analysis

Descriptive statistics were utilized for the initial categorization of the population. Logistic regression modeling was used for the main analysis. The outcome was thrombosis or not, and the main exposure was contraception. The variable estrogen-containing contraception was treated as an exposure to contraception for those subjects. Logistic regression was also used to determine the effect of severity, SCD genotype, and hydroxyurea. Confounders including body mass index (BMI) of >30, severity, smoking status, and central line status at any point were considered, and models with interactions between contraception ever and confounders were fitted using multivariate logistic regression. When looking at age at thrombosis, the Equality of Variances using folded F statistic found that the groups had differing variances; thus, a t-test using the Satterthwaite method was used. To determine differences in proportions of the groups in demographics, Chi-squared with Fischer’s exact test was used. Cox regression analysis was performed to compare the hazard of thrombosis among patients using estrogen-containing combined HC or progestin-only medications versus those not using HC. The analysis was adjusted for disease severity determined by the complex scoring system and for risk factors such as BMI, smoking status, age, and central line status. Statistical analyses were performed with GraphPad Prism (version 10) and IBM SPSS Statistics (version 29.0.1.0 [171]).

## 3 Results

We identified 370 women with SCD between the ages of 15 and 49 examined in the UNC system from 2010 to 2022 (Table 1). The average age at data extraction was 32.71 ± 7.75 years. Of these, 93 (25.1%) patients had a VTE recorded in the record. In our cohort, the average age at thrombosis was 27.13 ± 6.9 years. Of the 93 patients with VTE, 35 (37.6%) had DVT, and 41 (44.1%) had a pulmonary embolism. Other events included 3 portal vein thromboses, 1 clinically significant superficial thrombosis, 1 left atrial thrombus, and 1 left ventricle thrombus, and 11 had sites that were not listed.

**Table 1:** Characteristics of the patient cohort, including type of sickle cell disease, severity of disease, contraception status, and hydroxyurea status.

Demographics	Total	VTE	No VTE	*P* value
Number (%)	370 (100)	93 (25.1)	277 (74.9)	
Race	Black – 368 (99%)			
	Hispanic – 2 (1%)			
Age (y) (mean ± SD)	32.71 ± 7.75	27.13 ± 6.90^a^	32.50 ± 8.47	*P* < .001
Genotype (%)	SS: 225 (60.8)	SS: 67 (72)	SS: 158 (57)	*P* = .01^b^
	SC: 93 (25.1)	SC: 16 (17.2)	SC: 77 (27.7)	*P* = .05^c^
	Sβ+: 33 (8.9)	Sβ+: 6 (6.5)	Sβ+: 27 (9.7)	*P* = .40^d^
	Sβ0: 19 (5.1)	Sβ0: 4 (4.3)	Sβ0: 15 (5.4)	*P* = .79^e^
Severity (%)	Mild: 120 (32.4)	Mild: 5 (5.4)	Mild: 115 (41.2)	*P* < .0001^f^
	Moderate: 100 (27)	Moderate: 23 (24.7)	Moderate: 77 (28.2)	*P* = .59^g^
	Severe: 150 (40.5)	Severe: 65 (69.9)	Severe: 85 (30.7)	*P* < .0001^h^
Hydroxyurea usage (%)	277 (74.9)	84 (90.3)	193 (69.6)	*P* < .0001
History of central line (%)	99 (26.7)	52 (55.9)	47 (17.0)	*P* < .0001
BMI	27.13	26.9	27.2	*P* = .75
History of smoking (%)	84 (22.7)	23 (24.7)	61 (22.0)	*P* = .57

Percentages have a denominator of the total of the column. P values derived from Chi-squared with Fischer’s exact test.

BMI, body mass index; SC, heterozygous sickle cell, hemoglobin C; SS, homozygous sickle cell disease; Sβ+, sickle cell plus beta thalassemia; Sβ0, sickle cell beta thalassemia null; VTE, venous thromboembolism.

a Age at event.

b SS versus all others.

c SC versus all others,

d Sβ+ versus all others.

e Sβ0 versus all others.

f Mild versus moderate or severe.

g Moderate versus mild or severe.

h Severe versus mild or moderate.

**Figure 2:**
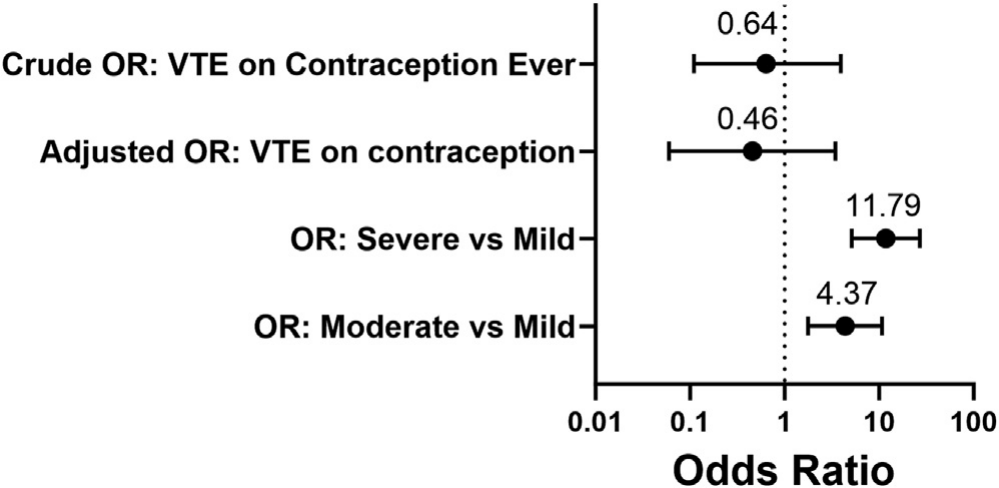
Oral contraception does not increase the risk of venous thromboembolism in women with sickle cell disease at this single-center site. Odds ratios (ORs) for several subgroups of analysis. The univariate analysis revealed a crude OR for venous thromboembolism on contraception was 0.64 (OR: 0.11-3.93; *P* = .63). Adjusted OR (for BMI, smoking status, severity, age, and central line status) was 0.46 (OR: 0.06-3.45; *P* = .45). When comparing severe to mild disease, the OR was 11.79 (OR: 5.14-27.06; *P* < .001), and moderate to severe was 4.37 (OR: 1.77-10.76; *P* < .001).

A noteworthy observation was that 219 (59.2%) patients utilized HC at some point. Among total HC users, 73 out of 219 (33.3%) experienced a VTE at any point, with 38 out of 184 (20.6%) experiencing VTE while actively using HC. In contrast, among the 151 patients who were never on HC, 20 (13.2%) had a VTE. The percentage of subjects that were exposed to HC in the VTE population was higher than the percentage in the non-VTE group (Table 1). Initial univariate linear regression analysis revealed an OR of VTE occurrence while being on all HC of 0.64 (95% CI: 0.11-3.93; *P* = .63). The adjusted OR, or multivariate analysis, which accounted for possible VTE confounders (BMI, smoking status, severity based on the complex severity score, age, and central line status) was 0.46 (95% CI: 0.06, 3.45; *P* = .45) (Figure 2)**.** It is worth noting that among the 93 cases of VTE, 30 instances were found to have a central line in place. However, establishing causation in these cases is a challenging task.

**Figure 3:**
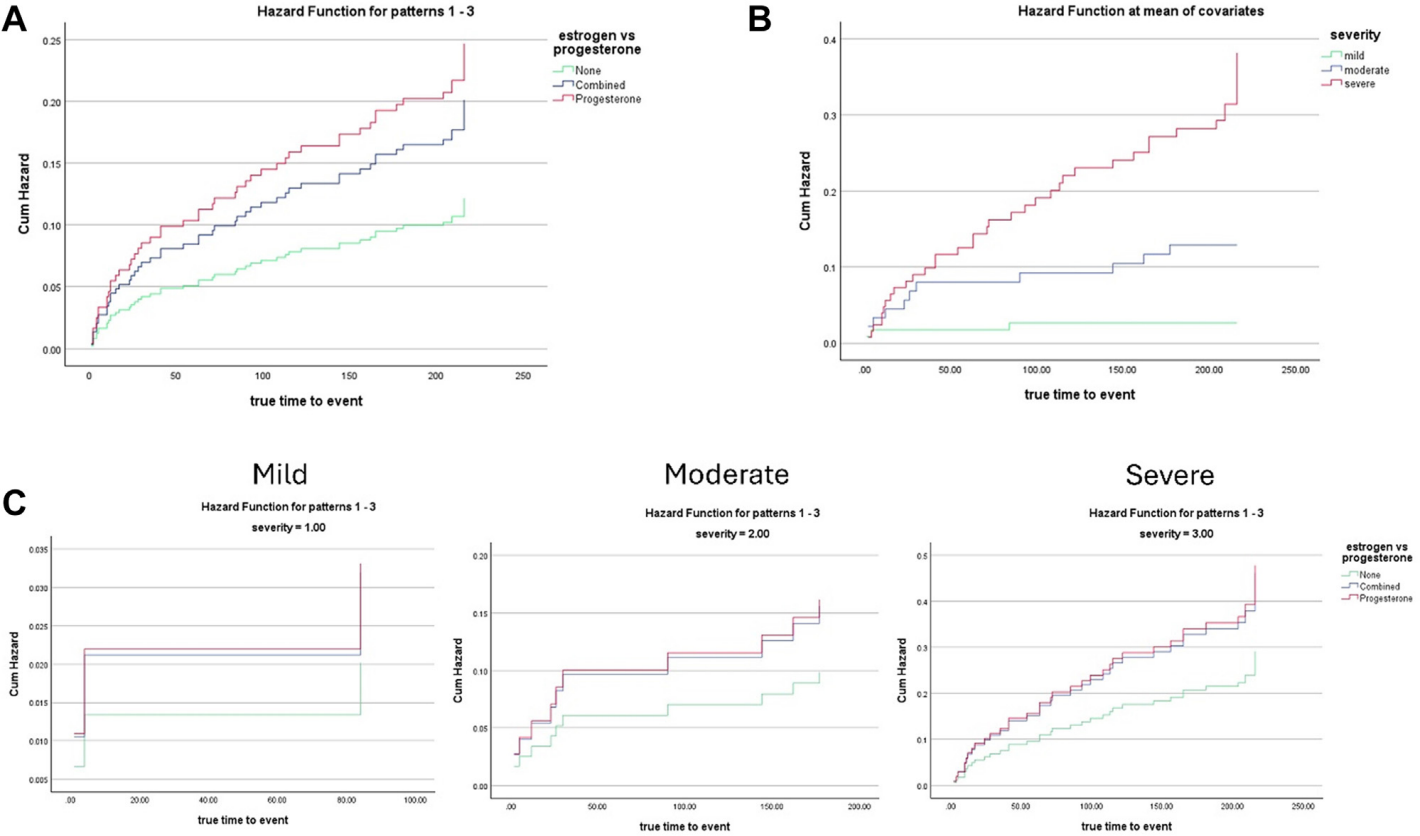
Cox regression analysis reveals that disease severity drives thrombosis risk. (A) hazard functions for estrogen versus progesterone versus no hormonal contraception. (B) Hazard functions of mild, moderate, or severe. (C) Hazard functions of estrogen or progesterone with adjustment for severity.

Interestingly, 35 of our patients had a history of HC usage that did not coincide with their VTE. Therefore, we excluded those patients for further analysis (Figure 1). In this group, we found that although 64 of 184 (34.8%) of the patients actively on HC were on estrogen-containing HC, only 10 of the 64 (15.6%) experienced VTE while taking estrogen-containing HC, and 120 (65.2%) experienced VTE while taking POC (Table 2). There were insufficient subjects on estrogen-containing HC to conduct logistic regression. This limitation also applied to all other individual types of POC (IUD, implant, DMPA, and POP); each group had too few subjects experiencing VTE while on contraception to perform logistic regression. Therefore, Cox regression analysis was performed to compare thrombosis hazards for patients on combined estrogen-containing HC or POC versus those not on HC. The time-to-event in this analysis was defined as the occurrence of thrombosis or the end of the study period. This unadjusted analysis yielded similar results to previous linear regression, with POC being a statistically significant hazard for thrombosis (HR: 2.03; 95% CI: 1.107-3.726; *P* < .05) (Figure 3A**)**. Considering the potential influence of disease severity on HC prescription strategies [31], we further adjusted our Cox regression analysis for SCD severity based on the complex model [36]. After stratification by severity (mild, moderate, severe), the association between POC and thrombosis risk was no longer significant (HR: 1.64; 95% CI: 0.894-3.018; *P* = .11) (Table 3), and that severe status, but not moderate, drove the risk (Figure 3B). After severity stratification, no difference in time-to-event hazard was observed between estrogen and POC treatment groups (Figure 3C–E**)**. Adjustment for individual VTE risk factors (smoking, central line status, age, and BMI) revealed no significant effects (Supplementary Data).

**Table 2:** Characteristics of the patients actively using hormonal contraception.

Category	Total	VTE	No VTE
HC, Active Use	184	38	146
Estrogen HC	64 (34.8)	10 (26.3)	54 (36.9)
POC	120 (65.2)	28 (73.7)	92 (63.1)
Severity	Mild: 140 (76.1)	Mild: 20 (52.6)	Mild: 120 (82.2)
	Moderate: 21 (11.4)	Moderate: 7 (18.4)	Moderate: 14 (9.6)
	Severe: 23 (12.5)	Severe: 11 (28.9)	Severe: 12 (8.2)

Percentages have a denominator of the active HC of their column. These data were used for Cox hazard analysis.

HC, hormonal contraception; POC, progesterone-only contraception.

**Table 3:** Results of Cox regression analysis when adjusted for severity.

Comparison	Hazard ratio (95%CI)	*P* value
Estrogen versus non-HC	1.54 (0.725-3.463)	.249
Progesterone versus non-HC	1.642 (0.894-3.018)	.110

HC, hormonal contraception.

The assessment of disease severity in this study was conducted by considering many factors in clinical care, as previously detailed [36]. In the VTE group, significantly fewer patients were categorized as mild, and significantly more patients were categorized as severe (Table 1). Therefore, logistic regression analysis was performed to compare severe or moderate disease against mild disease. The OR for severe versus mild was 11.79 (95% CI: 5.14-27.06; *P* < .001) and for moderate versus mild, it was 4.37 (95% CI: 1.77, 10.76; *P* = .001). In the simplified severity model that relied solely on the number of unplanned hospitalizations to classify patients, no statistically significant differences in OR for VTE were observed. Specifically, the OR for severe versus mild disease was 1.68 (95% CI: 0.55-5.08; *P* = .361) and for moderate disease versus mild, it was 0.79 (95% CI: 0.24-2.56; *P* = .69).

We conducted a comparison of VTE rates based on SCD genotype. In the total sample, 60.8% of patients were homozygous HbSS. Notably, there was a significant increase in the proportion of patients with HbSS genotype within the VTE group (72%) when compared with the no VTE group (57%) (*P* = .01) (Table 1). In a secondary analysis that distinguished severe genotypes of HbSS and SβThal^0^ from typically mild genotypes, HbSC and HbSβThal^+^, we did not find an increase in thrombosis rate with an OR of 1.61 (95% CI: 0.76, 3.41; *P* = .21). We also found that there were significantly more patients taking hydroxyurea in the VTE group (90.3%) compared with the non-VTE group (69.6%) (Table 1). However, the regression analysis showed no increase in the rate of VTE with regard to hydroxyurea use: OR of 1.73 (95% CI: 0.82, 3.55; *P* = .14).

## 4 Discussion

In this single-center cohort of female patients with SCD, we identified a high rate of VTE (25.1%) that occurred at an average age of 27 years. The incidence is higher than previously reported by Brunson et al. [8,18] (11.2%) and in the Cooperative Study of Sickle Cell Disease (11.3% by age 40) [10], but aligning with Roe et al. [19], who reported a rate of 24.6%. Our results describe a population of women with SCD who face a heightened risk of thrombosis, which occurs at a significantly younger age than described in the general population [8,10,38]. The primary objective of this study was to investigate the potential contribution of HC to the elevated risk of VTE in women with SCD. Both crude and adjusted OR remained nonsignificant, indicating that HC may not exacerbate the risk of VTE in this population.

At this institution, a noteworthy trend emerged regarding overall HC usage among those with SCD: 59.2% of the female SCD population had been prescribed HC. This exceeds the rates reported in previous studies [19,26,31,32,[39], [40], [41]]. Notably, 29.2% of the women taking HC in our study were on estrogen-containing HC, which is higher than initially anticipated since many providers advise against its use in women with SCD [26,28]. In our cohort, the patients taking estrogen-containing HC were equally distributed among mild, moderate, and severe disease categories. These findings are consistent with a recent retrospective analysis by Bala et al. [31], which examined a large Medicaid database. In their study of 27,950 women with SCD aged 12 to 44 years, 26% initiated new contraception claims, with 44.6% being prescribed estrogen-containing HC, indicating that this institution’s data align with broader trends. Moreover, their study found no difference in the thrombosis rates between individuals initiating estrogen-containing HC or POC. In their study, patients prescribed POC were more likely to be older, have more severe disease, and have a HbSS genotype. This indicates that disease status is considered when prescribing HC. Indeed, this is reflected in our study when adjusting for disease severity in both the linear and Cox regression analyses.

The results of this study indicate that using POC in women with SCD does not seem to increase the risk of VTE. This is supported by the linear regression, which did not show significance in terms of overall contraceptive usage, as well as by the hazard ratio, which normalizes when taking into account the severity of the disease. This contrasts with other prothrombotic conditions. For example, in cases involving FV Leiden mutations, the relative risk of VTE when taking estrogen-containing HC is synergistic, with an estimated OR of 20 to 30 for heterozygous patients and even higher for those with homozygous mutations [42,43]. Thus, POC is the preferred contraception strategy in these other patients with prothrombotic conditions because it does not significantly increase VTE risk [44]. It is important to note that the absolute risk of VTE differs significantly between FV Leiden and SCD, though with FV Leiden having approximately 16 events per 10,000 patient-years, while SCD is associated with roughly 5 events per 1000 patient-years [10]. This prompts consideration that SCD itself confers a higher risk of VTE compared with FV Leiden .

Consequently, it may be more challenging to discern a noticeable difference in VTE risk with the addition of HC in SCD patients, considering their baseline risk of VTE is already elevated. These disparities emphasize the immediate need to address thrombotic complications in women with SCD. Despite this elevated risk, this population has received less attention, and for that reason, we set out to determine other risk factors for VTE in our study population.

There is evidence that the severity of disease was the main driving factor for VTE in our population. This was supported not only by the logistic regression analysis which showed differences in disease severity but also by the Cox regression analysis which showed mitigation of effect when severity was factored in. Additionally, it was interesting to note that POC was found to have an increased hazard in the Cox regression, but its significance returned to insignificance upon adjustment for severity. This led us to theorize that higher-risk patients may be more likely to be prescribed POC at baseline.

In this study, a comprehensive disease severity classification that considers multiple factors and complications [36] was utilized and revealed significant differences between groups. The findings indicate that individuals with severe disease, as hypothesized, have an increased risk of VTE. The analysis of a simplified severity index, focusing solely on unplanned hospitalizations, did not reveal significant differences between groups. To validate these findings and better understand the factors contributing to VTE risk in this population, further research with larger sample sizes, including both retrospective and prospective studies, is necessary. This study emphasizes the need for a standardized and clinically validated system for quantifying disease severity.

### 4.1 Strengths and limitations

The strengths of this study included a detailed look at the diagnosis, confirmed comorbidities, and precise timing of VTE related to HC usage. Due to the multiple methods of confirmation of contraception and VTE presence, we could determine if patients were using HC at the time of the VTE events with more certainty than many other studies. Moreover, the overall size of the study was more than sufficient to determine the effect, with sample size calculations estimating about 148 needed to provide statistically significant effects.

The study has some limitations. We could not analyze the impact of estrogen-containing HC on VTE through logistic regression due to the limited number of cases where patients were using it at the time of VTE. Since the prescription rate of estrogen-containing HC is lower in women with SCD compared with the general population, our study is subject to a selection bias [26,28]. We were unable to consider all disease-specific complications and confounders to fully analyze the compounded effect of disease severity on HC utilization. Additionally, information on whether patients were on anticoagulation for other indications was not recorded. We could not differentiate between a patient’s history of central venous catheter and whether the catheter was in place at the time of VTE. To address this, we adjusted for catheter status as a general measure of disease severity and thrombotic risk.

### 4.2 Conclusions and future directions

Despite these limitations, our study adds to the inadequate data available on this topic in SCD patients. The study’s results thus highlight the unique considerations and risk profiles associated with contraception in women with SCD, underscoring the importance of tailored medical care for this patient population. Although the risk of HC was found to be insignificant overall, the incidence of baseline VTE is quite high. Our findings emphasize the significance of disease severity in conversations with women with SCD regarding contraception choices, as these factors appear to play a more substantial role in their potential thrombosis risk than HC. Clinicians should consider these findings and those previously published [19,31] when discussing reproductive care in women with SCD. It is still imperative to address the question of why women with SCD have a higher risk of VTE and if there is a synergistic effect between estrogen-containing HC and disease severity on VTE risk. To gain a more comprehensive understanding of the risk associated with estrogen-containing HC in individuals with SCD, future studies with larger sample sizes are warranted. We propose that contraception status is a critical data point to include in large nationwide patient databases to create more robust research resources. Further prospective and basic science studies are needed to understand the biochemical and physiological pathways that contribute to the pathology of VTE and identify biomarkers that may predict risk in women with SCD.

## Supplementary material

Supplementary Docx
