# Supplementary material for: Disease severity drives risk of venous thrombotic events in women with sickle cell disease in a single-center retrospective study
Source: Res Pract Thromb Haemost. 2024 Jun 10;8(4):102471. doi: 10.1016/j.rpth.2024.102471 (PMC11295566; doi:10.1016/j.rpth.2024.102471)
Supplement: Supplementary Docx [file mmc1.docx]

**Disease severity drives risk of venous thrombotic events in women with sickle cell disease: a single-center retrospective study.**

Light J, Abrams CM, Ilich A, Huang S, Zhu H, Baskin-Miller J, and Sparkenbaugh EM.

**Supplemental Methods and Data**

**Sickle Cell Disease and Venous Thromboembolism ICD 9 and 10 codes.**

SCD was defined by ICD 10 codes D571, D5740, D5700, or ICD 9 codes 282.61 and 282.62. Venous thromboembolism (VTE), including deep vein thrombosis (DVT) and pulmonary embolism (PE) were defined by ICD 10 codes I260, I269, I822, I823, I828, and ICD9 codes 415.x, 451.1, 451.11, 451.19, 451.2, 451.89, 453.x.

| **Results of Chi-Square analyses with different tests** | | | |
| --- | --- | --- | --- |
|  | Chi-Square | df | Sig. |
| Log Rank (Mantel-Cox) | 4.679 | 4 | .322 |
| Breslow (Generalized Wilcoxon) | 5.139 | 4 | .273 |
| Tarone-Ware | 5.044 | 4 | .283 |

**Multiple Confounders for VTE**

We performed a more comprehensive logistic regression that incorporated every risk factor for VTE defined by the American Heart Association. The resulting outcome did not significantly differ from that obtained when considering a representative set of confounders (BMI, smoking status, severity per complex severity score, age, and central line status). Upon adjustment for all of these risk factors, the odds ratio for thrombosis was 0.383 (95% CI 0.03 - 5.13, p=0.47), which is not significantly different from the adjusted OR reported in the manuscript.

| **Adjusted Cox Analysis** | | | |
| --- | --- | --- | --- |
| Adjustments for Risk of VTE on POC | p-value | HR | CI |
| Severity | 0.11 | 1.640 | 0.894 – 3.018 |
| Smoking Status | 0.058 | 1.891 | 0.979 – 3.650 |
| Central Line Status | 0.052 | 1.833 | 0.995 – 3.376 |
| Age | 0.035 | 1.983 | 1.051 – 3.743 |
| BMI >30 | 0.032 | 1.997 | 1.063 – 3.752 |

After adjusting for multiple factors (SCD severity, smoking, central line, age and BMI) the SCD severity remained the major confounder in progesterone-only treatment association with VTE.
